# 3D intraoral scanning techniques support the effects of crown morphology on dental caries

**DOI:** 10.1186/s12903-024-04292-9

**Published:** 2024-05-10

**Authors:** Jincheng Hao, Yuting Kang, Siyuan Wei, Jing Wang, Haiyan Wang

**Affiliations:** 1 Department of Stomatology, Beijing Chao Yang Hospital, Capital Medical University, No. 8 Gongtinan Road, Chaoyang District, Beijing, 100020 PR China; 2 Department of Thoracic Surgery, Beijing Chaoyang Hospital, Capital Medical University, No. 8 Gongtinan Road, Chaoyang District, Beijing, 100020 PR China; 3 Department of Stomatology, Ciqu Community Health Service Center of Tongzhou District, Beijing, China

**Keywords:** Dental caries, Tooth morphology, 3D intraoral scanning technology, Predictors, Oral health

## Abstract

**Background:**

With the development and utilization of three-dimensional (3D) intraoral scanning (IOS) technology, the morphological characteristics of teeth were quantitatively assessed. In this research, we aimed to explore the prevalence of dental caries in relation to each measurable morphological indicator of the tooth body via 3D intraoral scanning techniques.

**Methods:**

A hospital-based single-centre study was conducted at our hospital from Dec. 2021 to Apr. 2023. A total of 53 patients were involved in the study, providing complete morphological data for 79 teeth. Each patient completed an oral hygiene routine questionnaire and underwent examination by an experienced dentist to evaluate caries conditions before undergoing 3D intraoral scanning to obtain a digital dental model. Geomagic Studio 2014 was used to extract oral morphological data from the models. The acquired data were entered, cleaned and edited using Excel 2016 and subsequently exported to SPSS version 25.0 for analysis. Chi-square analysis and logistic regression analyses were employed to test the associations.

**Results:**

Among the participants, 33 (61.1%) were female, with a mean age of 26.52 ± 10.83 years. Significant associations were found between dental caries and the vertical distance between the distal tip and the gum (OR 14.02; 95% CI 1.80-109.07; *P* = 0.012), the distal lateral horizontal distance of occlusion (OR 0.40; 95% CI 0.18–0.90; *P* = 0.026), and the mesial horizontal distance of occlusion (OR 2.20; 95% CI 1.12–4.31; *P* = 0.021). The Hosmer–Lemeshow test indicated a *P* value of 0.33.

**Conclusions:**

The vertical distance between the distal tip and the gum, the distal lateral horizontal distance of the occlusion and the mesial horizontal distance of the occlusion were the influencing factors for dental caries (identified as independent risk factors). We hypothesize that these factors may be associated with the physiological curvature of teeth and the role of chewing grooves in plaque formation over time. However, further studies involving larger population samples and more detailed age stratification are still needed.

**Supplementary Information:**

The online version contains supplementary material available at 10.1186/s12903-024-04292-9.

## Introduction

The significance of preventing dental caries in contemporary society cannot be overstated. According to a report from the World Health Organization (WHO), the average incidence of permanent tooth caries worldwide is approximately 29%, representing more than 2 billion cases, confirming its status as one of the most common oral diseases worldwide. Moreover, untreated caries has emerged as the foremost chronic disease in children, affecting 514 million children worldwide [1, 2]. Caries, by eroding the body’s strongest material, tooth enamel, can cause severe pain and infections if left unattended, potentially necessitating costly surgical interventions and thereby imposing substantial societal and economic burdens [3–5]. According to statistics, the indirect costs of permanent teeth and deciduous teeth caries worldwide in 2019 were nearly $22 billion and $1.5 billion, respectively [2]. Therefore, early prevention and treatment of caries are of paramount importance. Identifying caries susceptibility factors aids in crafting novel frameworks for prediction indicators, enabling the formulation of targeted preventive strategies and health care policies. Currently, the known susceptibility factors for caries include the oral environment, personal oral hygiene, external dental fillers, and pathogenic infections. However, as one of the earliest and most intuitive types of oral data available to dental clinics and hospitals, tooth morphology data on the occurrence and progression of dental caries are rarely reported or studied. Establishing a correlation between the two could unlock immense potential, as systematic scanning and digitization of dental records would provide health care practitioners and researchers with an unprecedented resource, facilitating comprehensive analyses to refine diagnostic and treatment methodologies, especially amid the burgeoning era of big data and machine learning.

Previous studies have suggested a potential correlation between tooth morphology and caries [6–9]. As the most exposed part of the tooth, crown shape profoundly influences the occlusal relationship and tooth physiological function and is influenced by various environmental factors, such as lifestyle and dietary habits, potentially interlinking with caries [10–12]. However, due to the intricacies of tooth crown morphology, accurate depiction poses challenges, often because of the need to rely on prominent features as representative indicators. Previous studies on crown morphology and caries have focused mainly on the associations among gap type, convex type and caries [6, 9]. Advances in technology have led to the introduction of novel techniques to explore the associations between tooth morphological characteristics and caries. Optical coherence tomography, which can be used to obtain images of the internal structure and microstructure of tooth soft tissue, presents an avenue for caries detection and assessment of integrity [13]. However, due to limitations in the measurement accuracy of plaster, resin moulds and measurement technology, the morphology of dental crowns has not been quantified or accurately measured. Notably, no studies have investigated the correlation between dental crown morphology and caries. P Ntovas et al. also confirmed the feasibility of IOS technology for the diagnosis of caries in 2023. Their findings indicated no significant difference between the clinical visual examination and the on-screen assessment of the IOS, except for the sensitivity corresponding to caries in the inner half of the enamel [14].

In this study, we used 3D parameters obtained from IOS that objectively reflect the shape and occlusion of the dental crown. The 3D oral scan data of orthodontic patients from Beijing Chaoyang Hospital in Chaoyang District, Beijing, China, were used to evaluate the association between dental crown shape characteristics and caries incidence.

## Methods

### Ethics approval and consent to participate

The study was conducted in accordance with the Declaration of Helsinki (as revised in 2013) and was approved by the Ethics Committee of Beijing Chaoyang Hospital. As the study design was retrospective, the requirement for informed consent was waived, and the study design was approved by the appropriate ethical review committee.

### Patient screening

A cross-sectional study of oral scan data from 53 orthodontic outpatients who visited the North Ward of Chaoyang Hospital, Beijing, China, from December 2021 to December 2022 was conducted from September 2022 to April 2023 to assess the associations between local dental morphological characteristics and the prevalence of obvious caries. All the subjects had their first mandibular permanent molars erupted. The questionnaire was used to investigate personal oral hygiene habits, including the frequency of eating snacks aside from three meals per week, frequency of intake of drinks and fruit juices per week, consumption of plain water or gargling after eating, total number of brushing times in the past three days, seriousness of brushing, number of occasions of bleeding gums during brushing in the past seven days, whether toothpaste contains fluoride, number of times and when you use dental floss/interdental brush in the past week. Caries were then graded according to the International Caries Disease and Assessment System (ICDAS) by experienced and trained dentists. iTero Element 2, as a 3D oral scanner with fast scanning processing function, enhanced ergonomics and high-definition colour imaging technology, was used to collect 3D oral data for the subjects. The instruments were operated by a professional physician trained in the course. The scanning methods and precautions used were based on the “Full mouth Scanning Skills” provided by iTero. Individuals with severely compromised or absent dentition, where identifying anatomical integrity was impeded due to extensive tissue defects, extensive caries, inadequate cavity fillings, abnormal morphology, pronounced crowding, atypical positioning, or excessive tooth wear, were excluded from the study.

### Variable selection

The outcome variable of this study was whether or not obvious dental caries occurred. A positive result was defined as 1, and a negative result was defined as 0. Within the population studied, the mandibular first permanent molar emerges as the initial tooth to erupt in the permanent dentition. Given its predisposition to dental caries and its significance in dental pathology, our study concentrated on this particular tooth. To assess the mandibular first permanent molar, the primary anatomical characteristics considered were those pertaining to the crown’s exposure. These included the mesiobuccal cusp, distobuccal cusp, mesiolingual cusp, distolingual cusp, central fossa, mesiobuccal groove, distobuccal groove, lingual groove, and buccal ridge, among other relevant features. We used the quantifiable parameters as independent variables for statistical analysis, including the mesiodistal diameter of the teeth, the buccolingual diameter, the gingival margin location relative to the CEJ, the mesiobuccal cusp vertical height, the distobuccal cusp vertical height, the mesiolingual cusp vertical height, and the distolingual cusp vertical height. In particular, to simplify the analysis, only the deepest value of the dental crown surface groove was taken as one of the independent variables.

To evaluate the degree of mandibular first permanent molar occlusion, we used the angle malocclusion classification system to reflect the occlusal contact relationship of the upper first permanent molar and as a covariate reflecting the relationship between proximal and distal molar occlusion. Overjet and overbite were introduced as covariables to reflect the occlusal relationship of buccolingual diameter teeth. Since the upper first permanent molar has a larger tooth body and larger occlusal contact area, and the situation is more complicated than that of incisor teeth, the mesial and distal overjet and overbite were measured in this study.

### Data export

Upon logging into the physician’s account at myitero.cn, we navigated to the initial menu and selected the “patient” option to retrieve the roster of patient names. We then selected the desired patient’s entry to proceed to the order interface. We chose the entry designated “Invisalign iRecord” and subsequently clicked the “Export” option. In the subsequent pop-up window, we then chose “Open shell” from the “export type” column, set the data format to “single dental arch file (divided by occlusion),” and specified the file type as “STL (monochrome).” After ensuring that the “Hide patient name” option was unchecked, we then executed the export process by clicking the “Export” button.

### Data record

After opening the exported file and decompressing it, we used Geomagic Studio 2014 to open and import the ‘.stl’ file of the upper and lower dental arch. At that time, we were able to use the Geomagic Studio function to complete the measurement of dental anatomy features.

Measurement of the occlusal relationship: We selected both upper and lower arch models and created new objects from the selection to obtain an overall model of the upper and lower arches that retained the original occlusal relationship. By using the “Distance” option in the measurement option box in the analysis menu bar, we were able to measure the mesial and distal overjet and mesial and distal occlusion of the mandibular first permanent molar. At the same time, we were also able to determine the occlusal angle between the maxillary first permanent molar and the mandibular first permanent molar with the naked eye through model scaling.

**Fig. 1:**
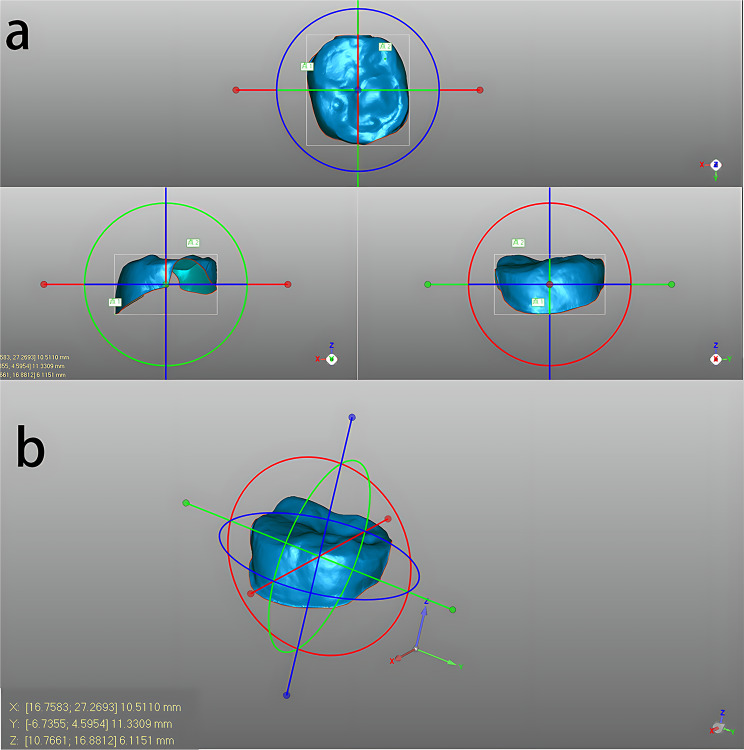
(**a**) When the “object mover” function was activated, the model was adjusted so that the x-, y- and z-axes were parallelized to the buccolingual diameter, mesiodistal diameter, and gingival tooth distance, respectively. (**b**) As the model was adjusted, the information at the bottom left represents each parameter we needed. The values of X, Y and Z represent the buccolingual diameter, mesiodistal diameter, and gingival tooth distance, respectively. In this case, the buccolingual diameter, mesiodistal diameter and gingival tooth distance were 10.5110 mm, 11.3309 mm and 6.1151 mm, respectively

**Fig. 2:**
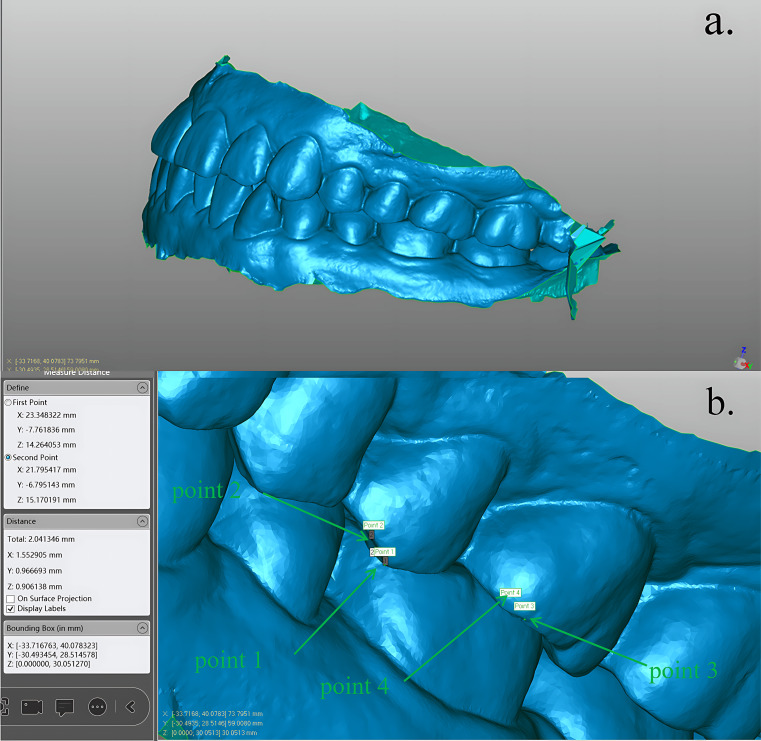
(**a**) In this view, we can clearly judge the angle malocclusion classification, and we can identify this example as angle I. **(b)** This picture was applied to the tooth, and the distance between the mesial and distal sides can be easily measured. The mesial distance can be acquired by metering between point 1 and point 2. The mesial-overbite length was 1.552905 mm (distance on X), and the mesial-overjet length was 0.906138 mm (distance on Z). The distal-overbite and distal-overjet lengths can be obtained by the same method that is metred between point 3 and point 4

Measurement of the anatomic features of the mandibular first permanent molar: We utilized the “Create Spline Boundary” function within the Boundary Options box of the Polygon menu bar to outline a closed polygon between the boundary circle of the first mandibular permanent molar and the gum. Subsequently, we isolated the tooth area, creating a distinct object for precise measurement of the selected tooth. To orient a separately created tooth, we employed the “Object Mover” tool from the menu bar, adjusting the model to align the buccolingual diameter with the X-axis, the proximal and distal median diameter with the Y-axis, and the gingival tooth diameter with the Z-axis (Fig. 1). Thus, the X-axis length, Y-axis length, and Z-axis length in the bounding frame dimensions corresponded to the buccolingual diameter, mesiodistal diameter, and gingival diameter of this tooth, respectively. Following the prompts within the bounding box, a “point” feature was added at the tooth’s lowest point to aid in identifying the precise positions of the mesiobuccal tip, distobuccal tip, mesiolingual tip, distolingual tip, and distolingual tip. Utilizing the “Distance” option in the measurement box of the analysis menu bar, we identified the maximum vertical distance from the lowest point of the tooth, corresponding to approximate positions of the mesiobuccal cusp, distobuccal cusp, mesiolingual cusp and distolingual cusp. These measurements were accurately recorded as the respective heights of the mesiobuccal cusp, distobuccal cusp, mesial cusp, and distolingual cusp. We located the position on the surface of the tooth crown with the minimum vertical distance from the lowest point, identifying it as the deepest point of the crown. Subsequently, we measured the vertical distance from this point to the highest point of the entire tooth, considering it to be the maximum depth of the crown. Occlusion information was also acquired in the same way (Fig. 2). For teeth for which all target variables could not be obtained due to missing structures, all measurable indicators were retained, and missing indicators were left blank.

### Caries scoring method and image source

**Fig. 3:**
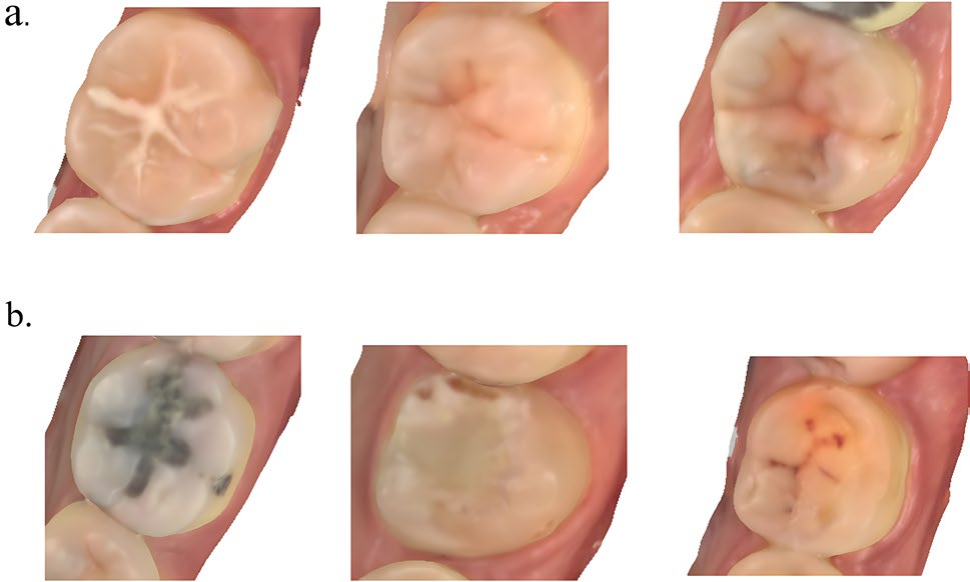
(**a**) This group of teeth was regarded as negative samples (tooth without caries). The conditions of the sealed tooth, normal tooth and slightly worn tooth are shown from left to right. (**b**) This group of teeth was regarded as positive samples (tooth with caries). The conditions of obviously decayed teeth, filled teeth and dentin eroded teeth are shown from left to right

This study used the ICDAS as the evaluation standard for caries. For the convenience of statistics and the accuracy of the discrimination of the results, ICDAS grades 0–4 were considered negative, representing no obvious caries, and were defined as ‘0’ in the data analysis; grades 5–6 were considered positive, representing obvious dental caries, and were defined as ‘1’ (Fig. 3). Visualization through a viewer on the MyiTero workstation and visual examination of the teeth by qualified physicians were used to accurately determine whether the observed object had obvious caries. Patients with severe caries (ICDAS grade 7), caries with complete loss of anatomical features or the absence of entire teeth were excluded from the study.

### Statistical analysis

We conducted scientifically rigorous calculations for determining the sample size. In accordance with FDA guidelines, the noninferior value cut-off was established at 0.25, which was derived from half of the lower limit of the 95% confidence interval of the positive control efficacy. Previous literature reported a 95% lower limit of approximately 0.25 for the incidence of dental caries in adults. This study’s noninferior threshold value was hence set at 0.25. Considering our centre’s caries incidence of 0.53, a unilateral α of 0.05, and a β of 0.2, PASS 14.0 software was used to calculate the required sample size of 59 patients, which was adjusted to 64 to accommodate a 10% potential loss to follow-up.

Descriptive analysis and normality tests were conducted to determine the distribution of participants’ demographic characteristics, personal oral hygiene habits and crown-related variables. Independent sample t tests were used to assess the prevalence of oral hygiene habits and caries in individuals in different age groups. The *P* results with assumed equal variance were selected when the Levin variance homogeneity test *P* > 0.05, and the *P* results with assumed unequal variance were selected when the Levin variance equality test *P* > 0.05. Logistic regression analysis was used to examine the associations of diet and hygiene habits and dental crown morphology with caries prevalence. The statistical significance level for all tests was set at 0.05, and all the data were analysed using SPSS 24.0 software.

## Results

### Patient general data

Data were available for 53 patients; a total of 106 teeth were scanned, providing 79 mandibular first molars that could be used for analysis. Fourteen teeth were severely decayed and therefore unrecognizable, 11 teeth lacked a clear occlusal relationship with maxillary molars that could not be measured, 1 tooth showed an abnormality distal to the buccal cusp, and the other tooth lacked the structure of the distal cusp, which led to their exclusion from the regression model. The survey participants who underwent clinical evaluation ranged in age from 9 to 50 years, with an average age of 26 years. There were 21 men and 33 women. The incidence of caries was 67.1%; that is, 53 teeth had ICDAS grade 5–6 caries. There were 44 teeth with angle-I occlusion, 20 teeth with angle-II occlusion, and 15 teeth with angle-III occlusion (Table 1). A total of 53 people received the questionnaire; 30 people completed the questionnaire, and 23 people did not respond.

**Table 1:** Baseline characteristics of patients(*n* = 53) used in our study

Variables	Mean ± SD
Age, y	26.52 ± 10.83
Gender, girls (%)	33(61.1)
The number of teeth used for analysis	79
The number of teeth with caries(%)	53(67.1)
**Occlusion angle**	
AngleI	44(55.7)
AngleII	20(25.3)
AngleIII	15(19.0)
Vertical dimension of distal buccal cusp/mm	6.175 ± 0.918
Vertical dimension of distal lingual cusp/mm	5.754 ± 1.221
Vertical dimension of mesial lingual cusp/mm	5.785 ± 1.179
Vertical dimension of distal cusp/mm	5.761 ± 0.992
Buccolingual diameter/mm	10.941 ± 0.542
Proximal distal diameter/mm	11.642 ± 0.633
Gingival margin location relative to the CEJ/mm	6.447 ± 1.030
Dental groove depth/mm	2.266 ± 0.659
Mesial overjet A/mm	2.787 ± 1.313
Distal overjet A/mm	2.628 ± 1.150
Mesial overjet B/mm	0.391 ± 0.923

### Basic description of tooth crown morphology by 3D intraoral scanning techniques and description of the 3D mouth scan operation

The vertical dimension of the distobuccal cusp was 6.175 ± 0.918 mm for all teeth, the vertical dimension of the distolingual cusp was 5.754 ± 1.221 mm, the vertical dimension of the mesiolingual cusp was 5.785 ± 1.179 mm, and the vertical dimension of the distal cusp was 5.761 ± 0.992 mm. The buccolingual diameter was 10.941 ± 0.542 mm, the proximal distal diameter was 11.642 ± 0.633 mm, the gingival margin relative to the CEJ was 6.447 ± 1.030 mm, and the dental groove depth was 2.266 ± 0.659 mm. The mesial overjet A was 2.787 ± 1.313 mm, which was slightly larger than the distal overjet A (2.628 ± 1.150 mm). The mesial overjet B was 0.391 ± 0.923 mm (Table 1).

### Differences in the oral habits of patients of different ages

Based on the average age of the population of 25 years, we divided patients ≤ 25 years into one group and patients > 25 years into another group. Using independent t tests, we found between-group differences (*P* < 0.05) in drinking plain water/gargle after eating, the frequency of brushing teeth in the last 3 days, the number of occasions of bleeding gums during brushing in the last 7 days, whether their toothpaste contained fluoride and the number of floss/interdental brush uses in the last 7 days. In all of these cases, the t value was negative, meaning that the values in the > 25-year group were greater than those in the ≤ 25-year group. That is, the > 25-year-old group drank plain water/gargle after eating more often, brushed their teeth in the last 3 days more frequently, used more floss/gap brushes in the last 7 days, experienced gum bleeding during brushing more often in the last 7 days, and used fluoride toothpaste more often than did the ≤ 25-year-old group (Table 2).

**Table 2:** Group differences in oral habits by independent T-test

Variables	Levene test *P*	t	*P*
Frequency of snacking outside three meals/week	0.948	−0.475	0.639
Frequency of beverage and juice intake/week	0.819	1.252	0.221
Drink plain water/gargle after eating	0.004	−4.165	0.001
How many times you brushed your teeth in the last 3 days	0.001	−2.384	0.026
Brushing seriousness	0.638	−0.124	0.902
Number of bleeding gums during brushing in the last 7 days	0.001	−3.305	0.004
Whether the toothpaste contains fluoride	0.827	−3.194	0.003
Number of floss/gap brushes used in the last 7 days	0.149	−2.394	0.023
Where do you use the floss/gap brush	< 0.0001	−1.559	0.132

* Drink plain water/gargle after eating: 1 = rarely or rarely, 2 = occasionally, 3 = often, 4 = every time; Brushing seriousness: 1 = only so-so, 2 = occasionally serious, 3 = mostly serious, 4 = almost always very serious; Whether the toothpaste contains fluoride: 1 = no, 2 = yes, 3 = don’t know; Where do you use the floss/gap brush: 1 = individual position, 2 = front teeth only, 3 = both front and back teeth, 0 = no use

* Homogeneity of variance: When *P* < 0.05 of Levene test, the result without assuming equal variance was taken, when Levene test *P* > 0.05, the result of assumed equal variance was taken

### Logistic regression for diet and hygiene habits, tooth morphology, occlusion and caries

Table 3 shows the logistic regression results for 79 teeth. The following variables were risk predictors of dental caries: the vertical dimension of the distal cusp (OR: 14.015; 95% CI: 1.801-1–09.068; *P* = 0.012), mesial overjet A (OR: 2.201; 95% CI: 1.124-4–0.311; *P* = 0.021) and distal overjet A (OR: 0.400; 95% CI: 0.178-0–0.896; *P* = 0.026). –The Hosmer–Lemeshow test showed that *P* = 0.33.

**Table 3:** Logistic regression for tooth morphology, occlusion and caries

Characteristics	OR (95% CI)	*P* value
Vertical dimension of distal buccal cusp/mm	0.158(0.017, 1.437)	0.101
Vertical dimension of distal lingual cusp/mm	2.726(0.351, 21.163)	0.338
Vertical dimension of mesial lingual cusp/mm	0.353(0.040, 3.143)	0.35
**Vertical dimension of distal cusp/mm**	**14.015(1.801, 109.068)**	**0.012**
Buccolingual diameter/mm	1.779(0.335, 9.460)	0.499
Proximal distal diameter/mm	0.569(0.170, 1.896)	0.358
Gingival tooth distance/mm	0.450(0.082, 2.478)	0.359
Dental groove depth/mm	0.773(0.328, 1.824)	0.557
Occlusion Angle		0.319
AngleI vs. AngleIII	0.761(0.175, 3.305)	0.716
AngleII vs. AngleIII	0.311(0.067, 1.436)	0.134
**Mesial overjet A/mm**	**2.201(1.124, 4.311)**	**0.021**
**Distal overjet A/mm**	**0.400(0.178, 0.896)**	**0.026**
Mesial overjet B/mm	1.776(0.879, 3.588)	0.11

* Hosmer-Lemeshow Test *P* = 0.333

Regarding the crown cusp, apart from the distal cusp (*P* = 0.012), the vertical dimensions of other mandibular first permanent molar cusps, including the distobuccal cusp, distolingual cusp and mesiolingual cusp, exhibited no significant associations with the risk of caries (*P* = 0.101, *P* = 0.338, *P* = 0.350, respectively).

With respect to the radial structure of the tooth, the buccolingual diameter, proximal distal diameter, gingival margin location relative to the CEJ and dental groove depth were not significantly associated with caries (*P* = 0.499, *P* = 0.358, *P* = 0.359 and *P* = 0.557, respectively).

In terms of the occlusion, there was no difference in caries between occlusion angle I and angle III (*P* = 0.716), and there was no difference in caries between angle II and angle III (*P* = 0.134). In regard to overjet, our study revealed that each 1 mm increase in mesial overjet A may increase the risk of caries by 1.101 times (*P* = 0.021), and each 1 mm increase in distal overjet A may reduce the risk of caries by 0.6 times (*P* = 0.026). However, we found no correlation between mesial overjet B and caries (*P* = 0.11).

We also accounted for diet and hygiene habits as potential confounders in our study and included hygiene habits in the regression model. However, both regression models, as detailed in Tables S1 and S2, revealed no significant associations.

## Discussion

The pathogenic factors of caries are complicated and have been well studied in the past, including the oral environment, personal oral hygiene, external dental fillings and pathogenic infections. The oral environment includes abnormalities in the quality and amount of saliva, inadequate fluoride exposure, gum atrophy, genetic factors, etc. Personal oral hygiene habits, such as frequent consumption of carbohydrate-rich foods and sugary drinks, increase the risk of caries [15]. High levels of caries occur in middle-income countries with high sugar consumption. Most caries occur in adults because the disease is cumulative. Additionally, caries are associated with socioeconomic status, with a greater incidence among impoverished and disadvantaged population groups [16, 17]. Moreover, factors such as dental fillers, orthodontic devices and poorly designed dentures contribute to the incidence of caries [18]. Although early colonization of *Streptococcus mutans* and other cariogenic bacteria in the oral cavity significantly contributes to caries development [19], the association between tooth morphology and caries remains relatively understudied.

In this study, based on the average age of the individuals in the study sample, the patients were divided into a > 25-year-old group and a ≤ 25-year-old group. There were differences in oral habits between the two groups, including whether the participants gargled after eating, the frequency of brushing their teeth in the past 3 days, the number of occasions of bleeding gums in the past 7 days, the severity of brushing their teeth, and the frequency of flossing in the past 7 days.

There are various studies on different aspects of the risk factors for dental caries. Saeed Bashirian et al. reported that in Iranian primary school students aged 7–12 years, age was significantly correlated with dental plaque, and the dental plaque index increased by 2.44 times each additional year [20]. N. Obregon-Rodriguez et al. showed that the risk factors for caries differed among children aged 12 and 15 years, and the prevalence of caries differed among children of different age groups and with different lifestyle habits [21]. Zeng et al. reported that children who were exclusively breastfed, had an increased frequency of daily snacking, a high frequency of bedtime snacking, and started brushing at a late age had an increased risk of caries, and the prevalence of caries increased with age, with the highest prevalence of caries occurring in the deciduous central incisors and deciduous molars [22]. These studies suggest that age and hygienic habits definitely influence the process of caries development. We believed that age, snack intake and the regularity of oral cleaning needed to be considered. Therefore, we included these factors as confounders. However, the abovementioned hygienic habits had no influence on caries in our patients after a series of statistical analyses, which allowed us to exclude them from the regression analysis. In the future, the regression algorithm can be used in a larger sample or in animal experiments to comprehensively analyse living habits, age, tooth morphology and caries prevalence to explore whether tooth morphology acts as a mediator or independent factor.

The tooth morphology is complex and difficult to describe precisely. The tooth crown morphology includes the tooth tip and its surroundings, the two faces of the ridge, the periphery of the fossa, and the sides of the groove. Among the surface structures of the first permanent molar, the vertical distance from the lowest point of the gingiva and occlusal surface can reflect the distribution of the tooth cusp, while the mesiodistal diameter and buccolingual diameter of the tooth reflect the size of the tooth. The angle I-III classification, along with the horizontal and vertical distances between maxillary and mandibular teeth, offers insight into the occlusal relationships of teeth. In addition, notable features include tongue grooves, buccal grooves, contact areas, interproximal spaces, embrasures, convexities of labial, buccal and lingual surfaces, incisal ridges, etc [23, 24]. Occlusal relationship features include occlusal classification (angle I-III) and overjet. In other studies, overbite, incisal path, inclination of incisal path, number of occlusal contact points and supporting cusps were also included as variables reflecting bite. Based on the role of tooth morphology in keeping the tooth surface clean, maintaining the stability of the dental arch and preventing food impingement, people have begun to consider the influence of tooth morphology on the occurrence and development of caries.

Previous studies on crown morphology and caries have focused mainly on the relationships among gap type, convex type and caries. As early as 1941, Klein and Palmer first studied the susceptibility of five different forms of teeth to dental caries. According to their research, the first permanent molars in the upper and lower jaw are the most susceptible to caries [25]. Next, Mark D. Macek and Eugenio D. Beltrdn-Aguila et al. further expanded the study and added a morphological permanent tooth type based on Klein and Palmer, showing that the prevalence of mandibular first permanent molars was still very high [26]. K R Ekstrand et al. reported that the morphology of interdental grooves was associated with the incidence of caries, which mostly occurred in the deepest part of the entrance to the grooves [6]. Leonor Sanchez-Perez et al. used a periodontal probe to assess crack depth and found that deeper cracks were more likely to cause caries (OR = 3.15, *P* = 0.028) [9]. D. W. Lewis et al. followed 98 children aged 11–15 years and reported a significant reduction in the incidence of dental caries in children who underwent pit and fissure sealing [27]. A Cortes et al. divided the tooth groove morphology into four categories according to the resin model: concave-concave, convex-concave, convex-convex and concave-convex. Teeth with concave-concave morphology were more likely to develop caries [28].

As technology advances, an increasing number of new techniques are being used to assess the associations between tooth morphological characteristics and caries. Optical coherence tomography can image the internal structure and microstructure of the soft tissue of a tooth and can be used for caries detection and integrity assessment [29]. Kyung Hyun Cho et al. used a portable device to evaluate quantitative light-induced fluorescence (QLF) and found that tooth surface fluorescence loss (ΔF) and bacterial activity (ΔR) can be used to predict caries [30]. Minh N Luong et al. used OCT to scan the occlusal surface of teeth, which is more sensitive to caries than X-ray examination combined with camera photography [31]. Keith Angelino et al. used nonionizing near-infrared light to detect demineralization and caries of enamel and established a 3D model. This technology has lower radiation exposure than cone beam projection computer reconstruction tomography (CBCT), which is commonly used in clinical practice, but it is also convenient and fast [32]. However, due to limitations in the measurement accuracy of plaster, resin moulds and measurement technology, the morphology of dental crowns has not been quantified or accurately measured. To date, no studies on the correlation between dental crown morphology and caries have been conducted.

With the development and application of IOS technology, we can scan and establish digital data on permanent tooth crown morphology, quantitatively evaluate tooth morphological characteristics for subsequent analysis, and more accurately explore the correlation between tooth morphology and caries. IOS operates through parallel confocal imaging technology, producing real-time 3D images of scanned objects and transforming them into surface polygon data representing teeth. Its benefits include rapid processing speed, minimal discomfort during scanning, cost-effectiveness, ease of data storage, and the ability to readily share data [33]. IOS is commonly used in prosthodontics, implantology, orthodontics and other fields [34]. Bohner et al. showed that IOS had more accurate results than did the desktop scanner in an abutment tooth model in vitro [35]. Its accuracy is also satisfactory in clinical practice. IOS can rival the scanning accuracy of short-span restorations (such as for a single tooth) of conventional print moulds. However, the use of IOS with the latest technology results in very little error in full dental arch restoration [34]. P Ntovas et al. verified the feasibility of 3D IOS for caries diagnosis. They found no significant difference between the clinical visual examination and the on-screen assessment of the 3D scan, except for the sensitivity corresponding to caries in the inner half of the enamel [14]. Therefore, it was feasible to judge the degree of tooth decay through the digital results of IOS, measure the morphological characteristics of a single tooth and analyse the occlusal relationship of multiple teeth. Building upon this information, we accessed the Beijing Chaoyang Hospital IOS database to obtain 3D parameters related to tooth crown morphology and occlusion. In our study, we found that the vertical distance between the distal tip and the gum (OR 14.02; 95% CI 1.80-109.07; *P* = 0.012), occlusal distal horizontal distance (OR 0.40; 95% CI 0.18–0.90; *P* = 0.026), and occlusal mesial horizontal distance (OR 2.20; 95% CI 1.12–4.31; *P* = 0.021) were significantly associated with caries. The correlation between dental crown morphology and caries was explored. The distal cusp, located at the junction of the buccal and distal surfaces of the teeth, is the smallest convex structure of the first permanent molar of the lower jaw. Its daily wear is the least, which can more truly reflect the initial state of the convex structure. Our research indicated a greater susceptibility to dental caries in individuals with a more prominent stature. Conventionally, deeper pits and fissures on the tooth crown surface, along with heightened surface convexity, were perceived to favour food debris retention and the proliferation of cariogenic bacteria. Furthermore, the presence of a distal cusp appears to influence tooth sensitivity to cariogenic substances. Due to the difficulty of cariogenic substances remaining on the convex structure and their tendency to accumulate in cracks, pits and fissures, the corrosion of lower enamel becomes more conspicuous. Consequently, the vertical distance between the distal cusp and the crown’s lowest point serves as an indicator of the tooth corrosion rate: a greater distance correlates with a faster corrosion rate. Overjet may affect tooth decay by affecting the occlusal relationship between the upper and lower teeth. In this study, we found that the overjet of the first pair of permanent molars has an impact on the occurrence of dental caries. However, interestingly, the mesial coverage value was a risk factor for dental caries, while the distal overjet value was a protective factor. We hypothesized that this may be because the deep mesial overjet does not facilitate the passage of food along the incisal path during chewing. It was also possible to trap food into the slit between the premolars and first permanent molars due to excessive movement while biting fibrous food. The occurrence of distal lateral overjet results from the close proximity of the second molar and the first molar, leading to a narrow crevice between the teeth. This limited space restricts food impaction and promotes a self-cleaning effect during chewing, potentially serving as a protective factor against caries. However, it is essential to acknowledge that the reasons behind the protective nature of distal overjet may be multifaceted. Further exploration is needed to uncover potential interactions stemming from the growth, development, structural and functional correlation between adjacent teeth, as well as the broader oral and maxillary system.

There were a few limitations to this study. First, our sample size comprised only 79 teeth with 12 variables, including 61 positive cases. Although our sample size met the logistic regression requirement that the total sample size be 5–10 times the number of independent variables, the sample size with an outcome of 1 should be 10 to 20 times the number of independent variables, and regular conclusions could not be drawn based on our regression results. Second, the selected features, such as the height of the tooth cusp and overjet, constituted only a partial scope of the dental crown features and were not comprehensive. Future studies should aim for larger, more diverse molar samples with a broader range of characteristic markers to mitigate potential biases. Third, during the utilization of computer software for sampling and measuring IOS outcome markers, there were discrepancies in tooth alignment. The buccolingual diameter, mesiodistal diameter and vertical diameter were parallel to the x-, y- and z-axes, respectively. This process needs to be completed manually based on human eyes; different people have different perceptions of “parallel”, and there are certain errors in manually adjusted “parallel”, which can introduce some errors for subsequent fine numerical measurements. However, despite these challenges, computer-based measurements yielded relatively more accurate results than resin models measured using a ruler.

The main strength of our study was the use of a 3D IOS, which can objectively reflect the three-dimensional parameters of crown morphology and occlusion, with a fast scanning speed, high accuracy and convenient process of collecting data, showing the possibility of providing a large amount of accurate data for early dental crown disease diagnosis in the coming era of big data medicine. For clinical dentists who deal with caries, performing a scan on a tooth without decay can indicate whether it is more susceptible to decay in the future. Initiating scans from the outset enables us to acquire a more comprehensive dataset of local patient morphology, enriching our database. Furthermore, dentists can obtain targeted caries predictions based on local databases. Orthodontists may benefit from our study because they can choose the appropriate orthodontic method based on the risk predicted. Patients deemed at greater risk may benefit from mobile appliances. Additionally, our study’s pioneering exploration of the relationships among tooth morphology, occlusal conditions, and dental caries represents a notable innovation. Our quantitative description of 3D crown morphology parameters for association analysis provides a novel perspective for understanding the aetiology and pathogenesis of caries. Furthermore, insights from our database can divergently inform denture design and tooth filling practices, potentially averting rapid wear and leading to improved prognoses.

## Conclusion

This research delineated methods for quantitatively describing tooth crown morphological characteristics and uncovering potential links between tooth crown structure and dental caries occurrence. Our findings mark the inception of a dental early warning system based on morphological assessments that integrates artificial intelligence for proactive tooth management. In our subsequent research endeavours, we aim to delve deeper into leveraging IOS, focusing on intricate structures such as pit and fissure evaluation and incisal path inclination, and further validating the association between dental crown morphology and caries. Additionally, our exploration with IOS will extend to assessing other maxillofacial conditions, including temporomandibular disorders (TMDs), periodontal diseases, and beyond.

### Electronic supplementary material

Below is the link to the electronic supplementary material.

Supplementary Material 1

## Data Availability

The data used and analysed in this study are included in the article or are available from the corresponding and first authors upon reasonable request.

## Abbreviations

3D: Three-Dimensional
IOS: Intraoral scanning
WHO: World Health Organization
CBCT: Computer reconstruction tomography
ICDSA: International Caries Disease and Assessment System
TMDs: Temporomandibular disorders
