# Supplementary material for: 3D intraoral scanning techniques support the effects of crown morphology on dental caries
Source: BMC Oral Health. 2024 May 10;24:549. doi: 10.1186/s12903-024-04292-9 (PMC11088106; doi:10.1186/s12903-024-04292-9)
Supplement: Supplementary file 1 — Supplementary Material 1 [file 12903_2024_4292_MOESM1_ESM.docx]

| **TableS1.** Logistic regression of diet and hygiene habits, tooth morphology, occlusion and dental caries | |
| --- | --- |
| **Characteristics** | ***P* value** |
| Vertical dimension of distal buccal cusp/mm | 0.989 |
| Vertical dimension of distal lingual cusp/mm | 1 |
| Vertical dimension of mesio lingual cusp/mm | 0.997 |
| Vertical dimension of distal cusp/mm | 0.989 |
| Buccolingual diameter/mm | 0.991 |
| Proximal distal diameter/mm | 0.997 |
| Gingival tooth distance/mm | 0.998 |
| Dental groove depth/mm | 0.994 |
| Occlusion Angle | 1 |
| AngleI vs. AngleIII | 0.995 |
| AngleII vs. AngleIII | 0.993 |
| Mesio overjet A/mm | 0.999 |
| Distal overjet A/mm | 0.998 |
| Mesio overjet A/mm | 0.994 |
| Frequency of eating snacks aside from three meals/per week | 0.988 |
| Frequency of intake of drinks and fruit juices/per week | 0.997 |
| Whether drinking plain water or gargling after eating | 0.988 |
| Total number of brushing times in the past three days | 0.987 |
| Seriousness of brushing | 0.993 |
| Number of bleeding gums during brushing in the past seven days | 0.996 |
| Whether toothpaste contains fluoride | 0.987 |
| Number of times you use dental floss/interfacial brush in the past week | 0.99 |
| When did you use dental floss/interfacial brush in the past week | 0.993 |
| * Hosmer-Lemeshow Test *P* = 1.000 | |
|  |  |

| **TableS2.** Logistic regression of diet and hygiene habits and dental caries | |
| --- | --- |
| Characteristics | P value |
| Frequency of eating snacks aside from three meals/per week | 0.78 |
| Frequency of intake of drinks and fruit juices/per week | 0.615 |
| Whether drinking plain water or gargling after eating | 0.6 |
| Total number of brushing times in the past three days | 0.053 |
| Seriousness of brushing | 0.223 |
| Number of bleeding gums during brushing in the past seven days | 0.078 |
| Whether toothpaste contains fluoride | 0.655 |
| Number of times you use dental floss/interfacial brush in the past week | 0.544 |
| When did you use dental floss/interfacial brush in the past week | 0.181 |
| * Hosmer-Lemeshow Test P = 0.016 | |
|  |  |
